# Inference for Nonlinear Epidemiological Models Using Genealogies and Time Series

**DOI:** 10.1371/journal.pcbi.1002136

**Published:** 2011-08-25

**Authors:** David A. Rasmussen, Oliver Ratmann, Katia Koelle

**Affiliations:** 1 Department of Biology, Duke University, Durham, North Carolina, United States of America; 2 Imperial College London, Department of Infectious Disease Epidemiology, London, United Kingdom; 3 Fogarty International Center, National Institutes of Health, Bethesda, Maryland, United States of America; Rega Institute, Katholieke Universiteit Leuven, Belgium

## Abstract

Phylodynamics - the field aiming to quantitatively integrate the ecological and evolutionary dynamics of rapidly evolving populations like those of RNA viruses – increasingly relies upon coalescent approaches to infer past population dynamics from reconstructed genealogies. As sequence data have become more abundant, these approaches are beginning to be used on populations undergoing rapid and rather complex dynamics. In such cases, the simple demographic models that current phylodynamic methods employ can be limiting. First, these models are not ideal for yielding biological insight into the processes that drive the dynamics of the populations of interest. Second, these models differ in form from mechanistic and often stochastic population dynamic models that are currently widely used when fitting models to time series data. As such, their use does not allow for both genealogical data and time series data to be considered in tandem when conducting inference. Here, we present a flexible statistical framework for phylodynamic inference that goes beyond these current limitations. The framework we present employs a recently developed method known as particle MCMC to fit stochastic, nonlinear mechanistic models for complex population dynamics to gene genealogies and time series data in a Bayesian framework. We demonstrate our approach using a nonlinear Susceptible-Infected-Recovered (SIR) model for the transmission dynamics of an infectious disease and show through simulations that it provides accurate estimates of past disease dynamics and key epidemiological parameters from genealogies with or without accompanying time series data.

## Author Summary

Reliable information about the demographic history of populations is important to both population biologists and epidemiologists, but is often absent or unreliable. There has therefore been great interest in developing statistical methods for inferring past population dynamics from gene genealogies reconstructed from molecular sequences. These “phylodynamic” methods take advantage of the fact that changes in population size can dramatically affect the shape of genealogies, making it possible to infer past changes in population size from a genealogy. However, in order for past population dynamics to be inferred, a demographic model must be specified. Current methods use demographic models that are often too simple for populations undergoing complex dynamics and generally do not allow for the parameters influencing the population dynamics to be estimated. We show how current phylodynamic methods can be extended to allow a much wider class of models to be fit to genealogies and illustrate our approach using an epidemiological model for the transmission of an infectious disease.

## Introduction

Epidemiologists increasingly rely on the ability to fit mechanistic models of disease transmission to data in order to estimate key parameters and elucidate the underlying processes driving disease dynamics. However, the nature of epidemiological data makes model fitting statistically challenging. Case report data such as time series of disease incidence are often incomplete or subject to severe biases like underreporting. Moreover, disease dynamics are generally only partially observed in that the exact times at which infection and recovery events occur are rarely, if ever, directly observed [1], [2], [3]. Researchers have therefore turned to the large amounts of molecular sequence data becoming available when case report data are insufficient. Gene genealogies can be reconstructed from sequence data and the times of coalescence events (i.e. branching events) can be used as a proxy for the timing of a subset of transmission events in the population. Using coalescent-based “phylodynamic” methods, it is then possible to infer the past dynamics of a disease from the lineages present in the genealogy, opening up the possibility of fitting models directly to genealogies.

Several coalescent-based methods for inferring past population dynamics from genealogies have already been developed [4], [5], [6], [7]. These methods employ the basic result of coalescent theory that the rate of coalescence is inversely proportional to the effective population size, *N_e_* [8]. Given the distribution of coalescence times over a genealogy, it is then possible to infer *N_e_*, which for an infectious disease is generally interpreted as an estimate of the number of infected hosts [9]. Past population dynamics can also be inferred by specifying a demographic model and fitting it to a genealogy [10], [11]. Most often, these demographic models are phenomenological and use simple parametric functions (e.g. constant size, exponential growth or logistic growth) or nonparametric functions that constrain population sizes to change smoothly or only at certain points in time [4], [6], [7]. Fitting simple parametric models like exponential growth to genealogies can provide insight into the epidemic dynamics of pathogens and provide estimates of epidemic growth rates and times of emergence [12], [13], [14]. Phylodynamic methods have also been applied to systems with far more complex endemic disease dynamics where the prevalence of the disease can fluctuate rapidly or undergo complex periodic oscillations. Remarkably, phylodynamic analyses of RNA viruses can sometimes recover features of their complex population dynamics due to a fast rate of sequence evolution and sampling of sequences over time [15], [16], [17].

While the vast majority of phylodynamic studies have inferred past dynamics by fitting phenomenological models to genealogies, a smaller body of work has investigated fitting mechanistic population dynamic models such as the well-known Susceptible-Infected-Recovered (SIR) class of models for the transmission dynamics of an infectious disease [9], [18]. Using mechanistic population dynamic models in place of phenomenological models may have major benefits. First, biologically important parameter values can be estimated along with past population dynamics, which can provide insights into the underlying processes driving historical population dynamics. For example, Pybus *et al.* [9] were able to estimate the basic reproductive number *R_0_* from viral genealogies for subtypes of Hepatitis C virus. Second, using these types of models should also improve our ability to correctly infer complex population dynamics, as they are constrained by population size trajectories that are dynamically feasible, rather than only biologically sensible (e.g., by being temporally continuous).

While the field of phylodynamics has made tremendous progress in recent years, methodological constraints limit the use of phylodynamic methods in epidemiological modeling more generally. First, only relatively simple epidemiological models where the number of infected hosts is a deterministic function of time can currently be fit using standard coalescent-based methods [4], [18], [19]. However, epidemiological dynamics are inherently stochastic and both demographic and environmental stochasticity can play important roles in disease dynamics [20], [21], [22]. Stochastic models are also essential for statistical inference since the variability, or over-dispersion, observed in real data can only be described statistically if stochasticity is included in the model [23]. This is especially true when fitting models to long-term data where the effects of stochasticity can accrue over time and cause the observed disease dynamics to deviate widely from the expectations of a deterministic model.

Current phylodynamic methods are also limited in that inference can only be conducted using genealogies. While phylodynamic methods will generally be used in the absence of historical data, other sources of data such as time series may be available alongside of sequence samples. This is especially the case for well-studied RNA viruses, where time series of case report data are collected as part of epidemiological surveillance programs. A number of statistical methods already exist for fitting mechanistic population dynamic models to time series data [1], [24], [25]. Generalizing such methods to fit mechanistic population models to genealogies as well would allow for inferences to be drawn from both time series and genealogies. Such an approach would allow for direct comparison between estimates derived from genealogies with estimates derived from time series data. Moreover, inference could then be conducted using both genealogical and population incidence data, potentially leading to more robust results.

The field of phylodynamics could therefore greatly benefit from having more flexible methods for genealogical-based inference. To this end, we have developed a general framework for phylodynamic inference that accommodates stochastic, mechanistic population dynamic models and can be integrated with other sources of data such as time series. In our framework, state-space models (SSMs) are used to model underlying biological processes mechanistically. While SSMs are already commonly fit to time series, we show how SSMs can also be fit to genealogies using coalescent methods. This allows for the model parameters and past population dynamics to be inferred from genealogies with or without accompanying time series data. Full Bayesian inference of all model parameters and past dynamics is performed using a method known as particle Markov Chain Monte Carlo (particle MCMC) [26], which uses particle filtering methods to fit SSMs to data without requiring an analytical likelihood function [23], [25]. This makes it possible to use a wide-class of SSMs for phylodynamic inference, including the stochastic, continuous-time dynamic models commonly used in epidemiology and population biology.

We present our approach by first briefly reviewing the fundamentals of SSMs and the particle MCMC method. We then present a stochastic SIR model for the dynamics of an infectious disease that we use throughout the paper as our SSM. For conceptual clarity, we first show how particle MCMC can be used to fit a SSM to time series data without a genealogy since this is a familiar problem in statistical inference. We then go on to show how the SSM framework can be expanded to include genealogies and how particle MCMC can be used to infer model parameters and past population dynamics from genealogies with or without accompanying time series data. Finally, we test our particle MCMC approach on simulated time series and genealogies. We find that reliable estimates of model parameters and past population dynamics can be obtained from time series data, a genealogy, or both. Moreover, we find that estimates obtained from genealogies approach the accuracy of estimates obtained from time series data when a large number of samples are collected serially over time.

## Methods

The general statistical framework we use to fit population dynamic models to either genealogies or time series data is based on state-space modeling. Structurally, state-space models (SSMs) consist of a process model and an observation model. The process model describes the underlying dynamics of the state variables 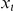 as a Markov process with model parameters 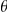 for all time points *t* in {1, …, *T*}:
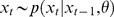
(1)Below, we use a SIR compartmental model [27] as the process model for the transmission dynamics of an infectious disease, with state variables being the number of susceptible (S), infected (I), and recovered (R) individuals. The exact state of the population at any given time (e.g., S_t_, I_t_, R_t_) is generally not observable. The state variables therefore remain unknown latent variables that must be inferred from available data. We therefore need an observation model to relate the observed data 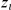 to the underlying process model:
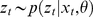
(2)For example, we will use an observation model that accounts for normally distributed observation noise in time series observations. While SSMs are typically used with time series data, here we use a more general approach where a coalescent model can be used in place of an observation model to relate a genealogy to the state variables in the process model.

To fit state space models to genealogical and/or time series data 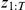, we use a Bayesian approach. Our primary goal is to find the posterior density of parameters 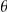 and latent state variables 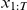:
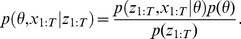
(3)From the posterior density, point estimates of model parameters as well as measures of uncertainty can be easily derived. However, for the models we consider here, the posterior density is analytically intractable. We therefore use an MCMC algorithm to sample from 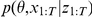 (for background on MCMC methods, see [28]). For illustrative purposes, we first present the following simple MCMC algorithm. Given current values of 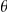 and 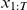, we:

**Step 1:** Propose new values for 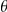 and 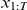 by sampling from the proposal density 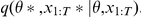

**Step 2:** Evaluate the posterior probability of 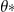 and 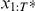 given 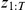, 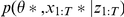, by computing 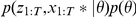.
**Step 3:** With probability
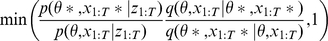
set 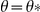, 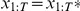 and 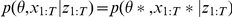; otherwise set 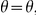 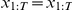 and 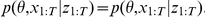

In practice, there are two major problems with using this naive MCMC approach. First, choosing an efficient proposal density for nonlinear and high-dimensional models is challenging [29]. Second, it is often difficult or impossible to evaluate the likelihood needed in step 2 when the disease dynamics are only partially observed through temporally aggregated data and the exact infection times are unknown [3], [30]. In our case, there is no analytical expression to impute over all unobserved infection times for continuous time, stochastic population models. We therefore use an approach known as particle MCMC [26], which employs a particle filtering algorithm to numerically construct an efficient proposal density without requiring that the likelihood be computed analytically.

The particle MCMC algorithm is essentially a particular version of the MCMC sampler presented above. While new values of 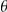 and 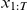 can be proposed together in Step 1, in particle MCMC new values for 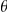 are first sampled from the proposal density 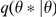 and then 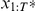 is independently proposed by sampling sequentially from 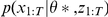, so that the proposal density has the form
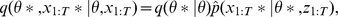
where 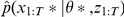 is a Monte Carlo estimate of 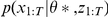 that must be obtained with a particle filtering algorithm (see below). The proposed 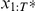 is therefore “adapted” to the data, which in our case, greatly improves MCMC efficiency [26]. As shown by Andrieu *et al.* [26], the acceptance probability in Step 3 is exactly given by
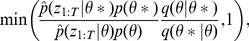
(4)where the Monte Carlo estimate 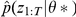 to the marginal likelihood is a byproduct of the particle filtering algorithm (see below). The full justification for using this acceptance probability is non-trivial, and we refer to [26]. We can therefore approximate the joint posterior density of 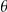 and 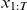 using particle MCMC, which would otherwise be very difficult or impossible using standard MCMC methods. Pseudo code for the complete particle MCMC algorithm is given in Text S1.

The particle filtering algorithm used in particle MCMC allows us to numerically approximate 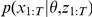 by simulating the unknown trajectories of the state variables from the process model (for reviews, see [31], [32]). The key idea behind particle filtering is to update particles sequentially through time so that at any time *t*, the weighted particles provide an approximation to the density 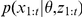. This is done by propagating particles forward from time *t-1* to *t* at each observation point in a two-step process. First, the state of each particle is updated by sampling new values from an importance density 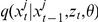, where 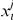 refers to the state of the *j*th particle at time *t*. Second, after the state of the particles has been updated, each particle is filtered according to the observation model and assigned a weight 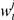. In general, the unnormalized particle weights are calculated as
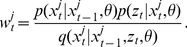
(5)In our case, there is no ideal importance density to sample from and particles are propagated by simulating directly from the process model [25], [31], [33], so that equation 5 simplifies to:
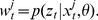
(6)In other words, the unnormalized weight assigned to a particle is simply the probability of observing the data *z*_t_ given the state of the particle as specified by the observation model. The unnormalized weights can then be summed to approximate the conditional marginal likelihood 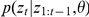,
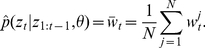
(7)By the law of total probability, an approximation to the marginal likelihood of the entire series of observations given 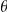 is simply
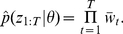
(8)This numerical approximation to the marginal likelihood is exactly the term that is required to evaluate the acceptance probability in equation 4 needed to perform MCMC sampling.

A common problem with particle filtering algorithms is that particle weights degenerate over time, meaning that most particles will carry little weight while a few will carry most of the weight [31], [32]. If this occurs, the particle system will not provide a good approximation to the density 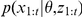. For long time series, this becomes a serious problem. The standard way of dealing with this issue is to resample particles from the population so that unpromising particles with low weights are not propagated forwards through time while promising particles are used to replenish the particle population [34]. We therefore calculate the normalized weights of each of the particles:
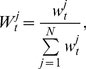
(9)and then resample particles according to their weights by multinomial sampling with replacement so that the total number of particles remains constant. Resampling is performed after every time step, after which particle weights are reset to 1/*N*. This particular particle filtering algorithm is known as bootstrap filtering and has the nice property that particle weights are independent of the particle's past trajectory [32], [33]. Note that without resampling, a proposal for 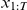 in each step of the particle MCMC algorithm can be obtained simply by sampling a single particle trajectory 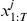 from the particle filter approximation to 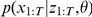. However, because particles are resampled at each time step in the particle filter, we have to track the ancestry of particles so that a single trajectory representing the path of a single particle through state space can be sampled. Pseudo code for the full particle filtering algorithm with resampling is given in Text S1.

### Inference with time series data

We first consider fitting a SSM to time series data 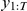 using particle MCMC. As our process model, we use a Susceptible-Infected-Recovered (SIR) epidemiological model with noise arising from variability in the transmission rate due to environmental factors. Using the Euler-Maruyama method, we can simulate this model forward in time with equations:
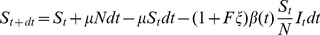
(10a)

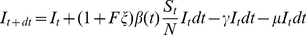
(10b)

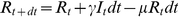
(10c)where 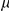 is the host birth/death rate, 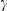 is the rate of recovery, 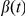 is the seasonally varying transmission rate, and *N* is the constant population size of the host, which we assume is known. We let the transmission rate vary sinusoidally with strength of seasonality 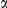: 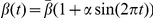 where the mean transmission rate is given by: 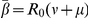 and 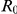 is the basic reproduction number. The noise term 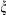 is given by 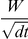, where *W* is a normal random variate with mean equal to zero and variance equal to one [35]. The constant *F* scales the magnitude of environmental noise. Along with equations 10a–c, we simulate a compartment, *C*, that tracks the cumulative number of infected individuals over time (i.e. the cumulative incidence):
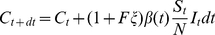
(10d)From *C*, we can compute the number of new infections occurring between any two time points *t*-1 and *t*: 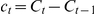. Assuming that only a fraction 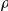 of these new cases are observed, and that observation error is normally distributed, the likelihood of observing *y* cases at time *t* is given by the observation model:
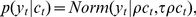
(11)where the mean is given by 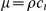 and the observation variance is given by 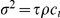, which depends on a scaling parameter 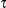, as in Ionides *et al.* [25].

Adapting the particle MCMC algorithm described above to fit the SIR model to time series data is straightforward. In the particle filtering algorithm, particle trajectories are simulated from equations 10a–d with process noise, so that each particle has a simulated incidence value 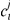. Particle weights are assigned using the observation model given in equation 11, so that unnormalized particle weights are calculated as
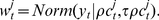
(12)Particle MCMC can then be used to sample from the posterior density 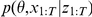. Here, 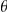 contains all the parameters in the SIR model as well as the observation model parameters 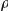 and 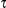. We can infer the trajectory of any of the state variables but we limit ourselves to inferring 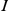 so that 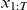 stands in for the number of infected hosts from *t* = *1* to *T*. Likewise, the initial conditions for all the state variables could also be inferred but we do not estimate them here since they are known values in the mock data we use to test the algorithm. Technical details on the implementation of the particle MCMC algorithm used to fit the SIR model are given in Text S2.

### Inference with a genealogy

We now turn to using particle MCMC to infer model parameters and latent variables from a genealogy. For illustrative purposes, we will use the same epidemiological model as detailed above. To see how a genealogy can be used to reconstruct the past population dynamics of a disease, first imagine that every infected individual is included in an infection tree with branching times that correspond to transmission events and tip times that correspond to recovery events. In this hypothetical case, the past prevalence of the disease at any time would simply be the number of lineages present in the genealogy at that time and the likelihood of the genealogy under a given population dynamic model could easily be computed since the times at which infection and recovery events occur would be known. In reality, we cannot observe the complete genealogy but we can reconstruct a partial genealogy from sequences sampled randomly from infected individuals over time. Coalescent theory provides us with the necessary probabilistic relationship between an incomplete genealogy and the underlying population dynamics 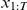 needed to fit a SSM to a genealogy of randomly sampled individuals. Specifically, the coalescent model will allow us to calculate the likelihood of observing a certain genealogy given the population dynamics 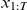, just as the observation model allowed us to calculate the likelihood of time series observations given 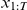.

Under the standard neutral coalescent model, the times between coalescent events in a genealogy are exponentially distributed so the probability of observing a coalescent event after time *t* is
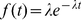
(13)where 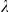 is the rate of coalescence. For many population models, the rate of coalescence depends on the number of lineages present in the genealogy *i*, the effective population size *N_e_*, and a factor 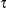 that rescales generation time into calendar time, so that
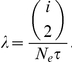
For an infectious disease, *N_e_* depends on the number of infected hosts *I* and the variance in the number of secondary infections an infected individual causes. Genealogical time has generally been rescaled into calendar time by defining the generation time scaling factor 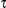 as the duration of infection [4], [19]. However, for an epidemiological model like the SIR model, the generation time of the disease is more appropriately defined as the average length of time it takes an infected individual to infect a susceptible host. Under our SIR model, the generation time is not constant over time since it depends on the rate at which infections occur, which is equal to 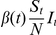. There is therefore no linear relationship that can be used to rescale genealogical time into calendar time. We therefore follow Volz *et al.* [18], and write the rate of coalescence under our SIR model as:
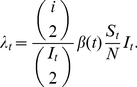
(14)Equation 14 has the intuitive interpretation that the rate of coalescence is equal to the overall rate of transmission in the population multiplied by the probability of observing a transmission event in the sample fraction, which is given by the ratio of the two binomial coefficients in the leading term. In practice, we round 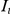 to its nearest integer value when computing the rate of coalescence so that 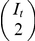 is always computable.

The exponential probability density function given in equation 13 can be combined with the expression for the rate of coalescence for an SIR model given in equation 14 to calculate the likelihood of the waiting time between any two coalescence events as a function of the state variables in the SIR model. The total likelihood of a genealogy can therefore be obtained by dividing the genealogy into coalescent intervals and taking the product of the likelihoods over all coalescent intervals. However, to enable comparison with inference using time series, the genealogy must also be partitioned at intervals that correspond to the observation times {1∶*T*} in the time series. Each of these time intervals is further divided into subintervals of size *dt*, where *dt* is the time step used in the simulation of the process model, given by equations 10*a*–*c* above. We assume that these *dt* subintervals are sufficiently small so that the number of infected and susceptible individuals does not significantly change within a subinterval. This assumption makes the rate of coalescence constant within subintervals, allowing us to use the exponential density given in equation 13 to compute the likelihood of the genealogy over these short subintervals. In addition to these intervals and subintervals, we allow for the general case that sequence data are sampled serially over time (i.e., the genealogy is heterochronic), such that, altogether, there are four types of time points which divide the genealogy into temporal sections: ‘observation’ time points 1∶*T*, time points every *dt* between these time points, sequence sampling times, and times at which lineages coalesce. The main difference between using a genealogy instead of time series data is that the observed data *z*_t_ are now the vector of time subintervals ***ω***_t_ between two observation time points *t*-1 and *t*, created by the *dt* time points, the sequence sampling times, and the coalescent times, rather than time series counts *y*_t_.

To compute the likelihood of the genealogy over a given time interval 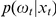, we can first write it as a joint probability of observing each subinterval time:
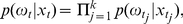
(15)Here *j* indexes the subinterval, and *k* is the number of subintervals in the observation time interval ending at time *t*. The likelihood of observing a subinterval time 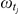 is simply given by equation 13 above if the subinterval ends in a coalescent event:
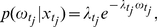
(16)where 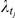 is the instantaneous rate of coalescence at time 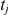, which can be computed from the values of the state variables in 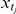 using equation 14. If the subinterval does not start at a *dt* partition time, but instead at a coalescent event or a sampling event, 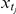 are the state variables at the closest *dt* partition time in the future.

The probability of observing subinterval time 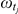 if subinterval *j* does not end in a coalescent event is given by the probability that a coalescent event has not occurred within this time period is:
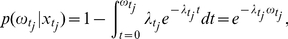
(17)as first described by Rodrigo and Felsenstein [36]. In the context of particle MCMC, the likelihood of the genealogy over the observation interval given by equation 15 is used to weight each particle at observation time *t* as described above.

### Inference with both time series and a genealogy

Finally, we show how model parameters and past population dynamics can be inferred from both time series data and a genealogy together with particle MCMC. As before, we use the epidemiological model provided by equations 10. The joint likelihood of observing both the time series and the genealogy in the time interval between *t-1* and *t* is given by:
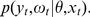
(18)Assuming that the genealogy is independent of the time series data, this joint likelihood can be re-written as:
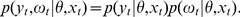
(19)Independence can be assumed if the samples in the genealogy are drawn from the infected population independently of which infected hosts are counted in the time series data. This is generally not the case, as the samples present in the genealogy are usually taken from a subset of infected hosts who are counted in the time series data. However, in our case, the fraction of infections counted in the time series data and the fraction present in the genealogy are both chosen at random. Therefore, the joint likelihood of observing both sets of data at time *t*, given the model and parameters 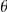, is given by the product of equation 11 and equation 15. In the context of particle MCMC, the unnormalized weight assigned to each particle is then the joint likelihood given in equation 19.

## Results

**Figure 1:**
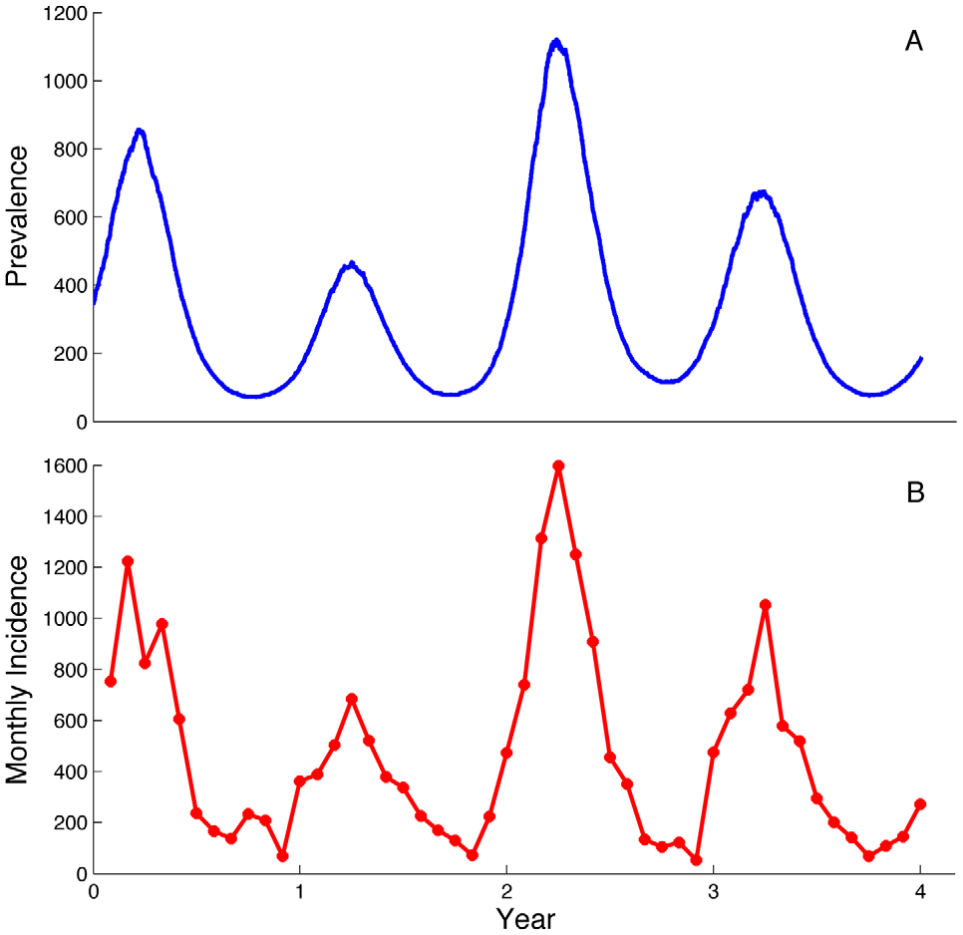
Simulated infection dynamics and time series used to test the particle MCMC algorithm. (**A**) Disease dynamics (*I*) obtained by simulating from the SIR process model (equations 10) over a 4-year period. (**B**) Corresponding time series of monthly incidence reports simulated from the observation model (equation 11). Parameters used in the simulation of the process model were: 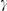 = 3/month, 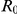 = 10, 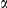 = 0.16, and *F* = 0.012. Other process model parameters that were assumed to be known were: 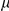 = 0.0017/month, and *N* = 5 million. Parameters used in the simulation of the time series data were: 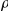 = 0.43, and 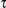 = 15.

**Figure 2:**
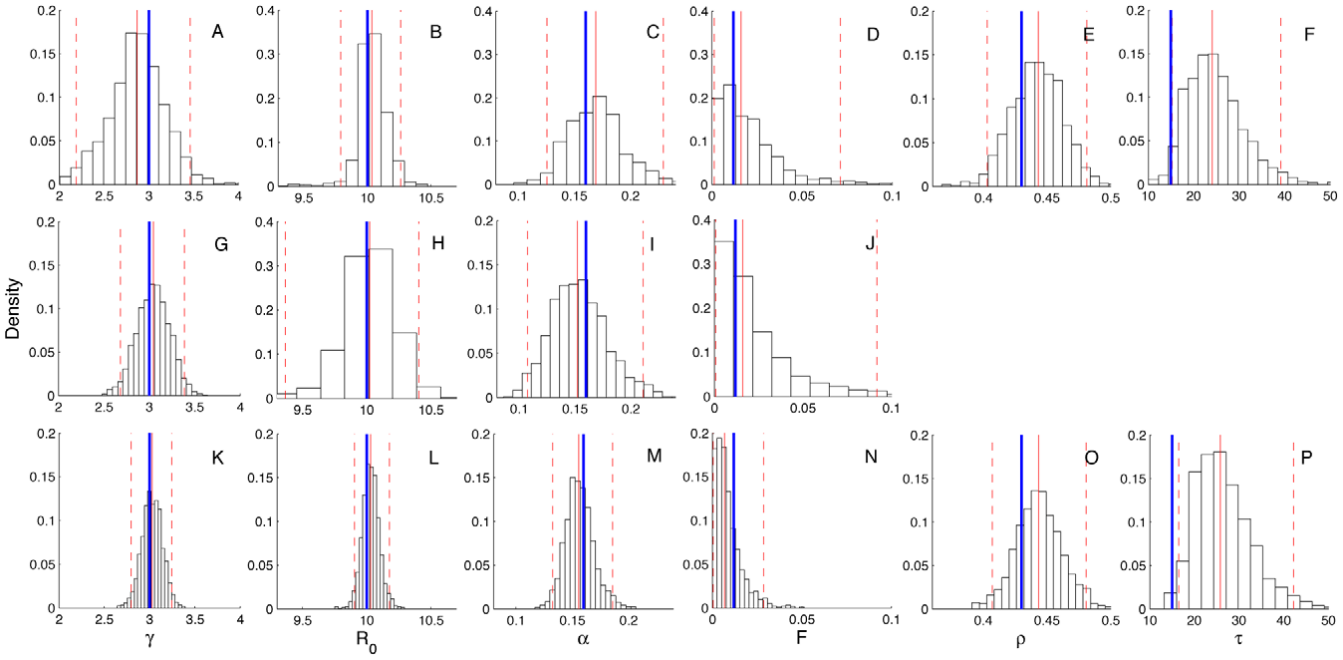
Posterior densities of estimated model parameters. Frequency histograms representing the marginal posterior densities of the SIR model parameters obtained using the particle MCMC algorithm. Vertical blue lines are placed at the true values of the parameters, solid red lines are the median value of the posterior densities and dashed red lines mark the 95% Bayesian credible intervals. From left to right, the parameters are the recovery rate 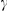, the basic reproduction number 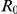, the strength of seasonality 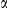, the parameter scaling the strength of environmental noise *F*, the reporting rate 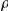, and the observation variance 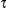. (**A–F**) Parameters inferred using time series data. (**G–J**) Parameters inferred using a genealogy. Parameters 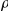 and 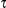 cannot be inferred using only a genealogy because they are parameters associated with the time series observation model. (**K–P**) Parameters inferred using both a genealogy and time series.

**Figure 3:**
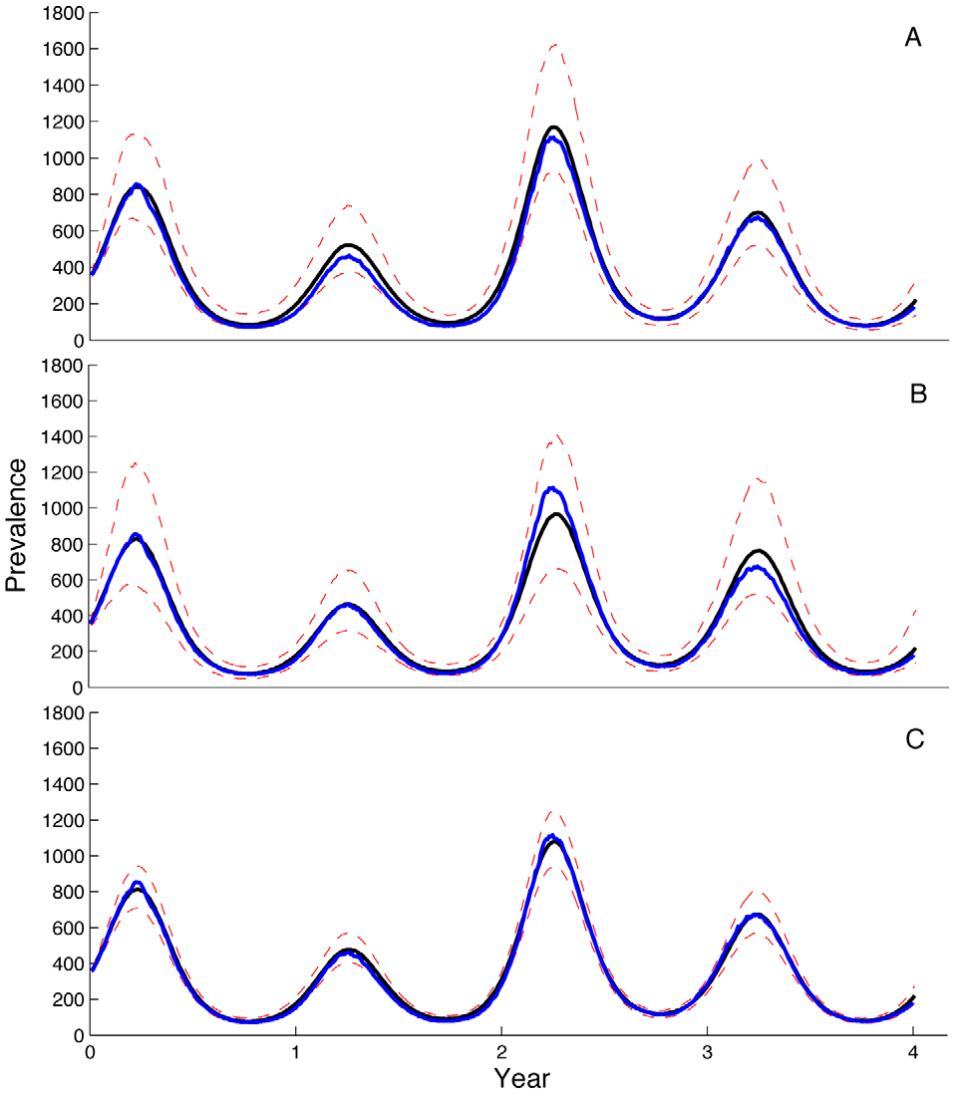
Posterior densities for disease prevalence over time. Series of posterior densities for disease prevalence *I* over time obtained using particle MCMC. Blue lines represent the exact simulated prevalence, black lines are the median of the posterior density and dashed red lines represent the 95% credible intervals. (**A**) Prevalence inferred from time series data. (**B**) Prevalence inferred from a genealogy. (**C**) Prevalence inferred from both a genealogy and time series.

To illustrate the ability of the particle MCMC algorithm to estimate model parameters 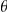 and the latent state variables 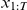 from time series data, we simulated a mock dataset using the SIR process model. Figure 1A shows the simulated dynamics of the latent variable *I* over time. Figure 1B shows the mock incidence data 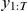 that are drawn using simulated *c* values (i.e. the cumulative incidence) and the distribution given in equation 11 to add normally distributed observation noise. The posterior densities of the process and observation model parameters inferred from the mock time series are shown in Figure 2A–D. As shown, the algorithm provided accurate estimates of the SIR process model parameters, with the true parameter values generally falling well within the 95% Bayesian credible intervals (CI). For the parameters of the observation model, we were able to accurately estimate the reporting rate 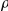 but found the observation variance 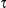 more difficult to estimate (Figure 2E–F). The series of posterior densities for the latent variable *I* (i.e. the prevalence of the disease over time) show that the algorithm accurately estimated the dynamics of latent variables (Figure 3A). The wider CI for the prevalence during seasonal peaks in prevalence relative to the offseason reflects the fact that environmental noise scales with the rate of transmission in our model, which is larger when prevalence is high.

**Figure 4:**
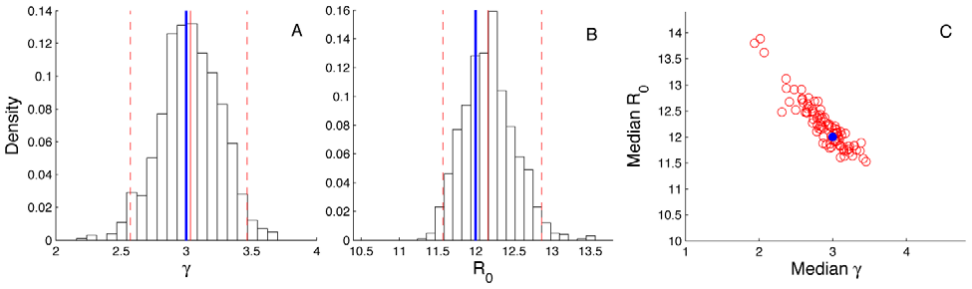
Posterior densities of parameters under epidemic conditions. Posterior densities of the parameters 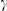 and 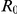 estimated from 100 independent genealogies obtained from simulated epidemic dynamics. (**A–B**) Frequency histograms representing the marginal posterior densities of 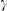 and 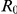 obtained from a single representative simulation. (**C**) The distribution of the median values of the posterior densities of 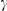 and 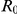 in parameter space for all 100 simulations (open red circles). The solid blue circle marks the true values of the parameters. Note that in our model formulation, 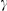 and 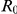 are independent parameters, with the transmission rate computed as 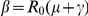.

We also tested the ability of the particle MCMC algorithm to infer parameters and past dynamics directly from genealogies. We obtained mock genealogies from our population dynamic simulations by tracking the ancestry of infections in the population and recording times at which infection and recovery events occurred. A subset of infection lineages were then randomly sampled at random times and their ancestry traced backwards through time so that transmission events correspond to coalescence events among the sampled lineages. We first checked if the coalescent model could be used to provide accurate and unbiased estimates of epidemiological parameters from genealogies. To check for possible biases, we tested the algorithm using epidemic dynamics with parameter values that lead to an epidemic unfolding and ending within a 12-month period. The shorter length of these simulations allowed us to check the performance of the algorithm using genealogies obtained from simulating the epidemic dynamics 100 times. As can be seen from Figure 4A–B, the epidemiological parameters 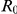 and 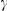 could be accurately estimated from the genealogies. However, we found it difficult to estimate the environmental noise term *F* from genealogies over such a short time period, so we fixed *F* at its true value. Figure 4C shows the distribution of the estimated median values of the posterior densities of 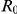 and 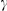 in parameter space for all 100 simulations. In spite of the strong negative correlation between these two parameters, the estimates cluster around the true parameter values, with the true values of 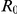 and 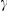 falling within the estimated 95% credible intervals 90 and 92 times out of the 100 simulations, respectively.

**Figure 5:**
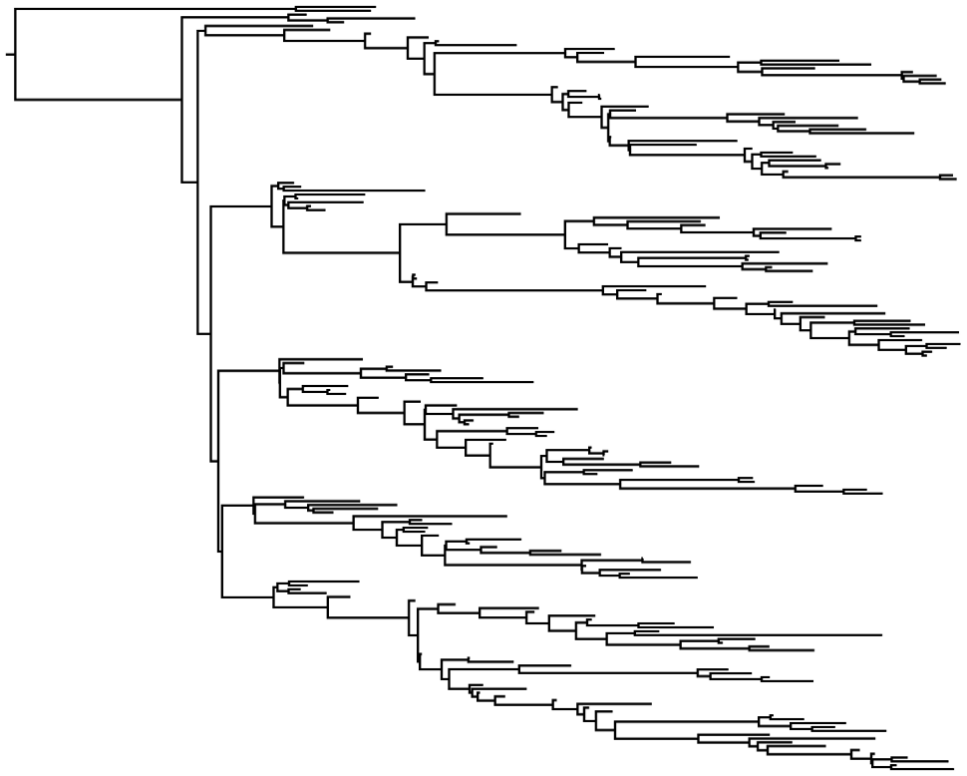
Simulated genealogy used to test the particle MCMC algorithm. Genealogy obtained from the simulated disease dynamics shown in Figure 1A. The genealogy contains 200 terminal nodes corresponding to sequence samples being collected sequentially over time with yearly sample sizes of approximately 50 sequences. Sampling events were chosen to occur at random times over the entire interval of the times series.

Since we were able to obtain accurate parameter estimates from genealogies under simple epidemic conditions, we next tested the ability of the particle MCMC algorithm to estimate parameters and latent state variables from genealogies under more complex population dynamics. To do this, we generated a mock genealogy containing 200 terminal nodes from the same population dynamic simulation shown in Figure 1. The mock genealogy is shown in Figure 5. The posterior densities of the process model parameters inferred from the mock genealogy show that our method could accurately recover the values of the epidemiological parameters (Figure 2G–J). The series of posterior densities for the latent variable *I* over time likewise show that our method can accurately estimate past disease dynamics from a genealogy (Figure 3B). This is highly encouraging, as it suggests that both model parameters and past population dynamics can be accurately estimated from a genealogy even in the absence of any time series data as long as the number of sequences sampled over time is sufficiently large.

Although the credible intervals for the process model parameters and past disease dynamics are wider when using the genealogy than when using the time series data, the width of the credible intervals likely depends heavily on the sampling effort. We therefore investigated a range of sample sizes to explore how different sample sizes affect the accuracy of and uncertainty associated with our estimates. Summary statistics for the posterior densities of the parameters and past prevalence of the disease are given in Table 1. Even with small sample sizes, reasonable estimates were obtained and the loss of accuracy in estimating parameters was most likely due to the difficulty of estimating the environmental noise term *F*, which is strongly correlated with other parameters, when the sample size was small. If the sample size is initially small, including more samples dramatically improves the accuracy and reduces the level of uncertainty in parameter estimates. However, going from an intermediate number of samples (∼100–200) to a large number of samples (∼400) does not dramatically improve estimates, suggesting only a moderate amount of sequence data is required for accurate inferences to be drawn from genealogies. Similar results were obtained for estimates of the past prevalence of the disease. We quantified the effect of including more sequence data by computing the root mean squared deviation (RMSD) of the inferred median of the posterior densities of disease prevalence from the true prevalence values. Increasing the number of samples initially reduces the RMSD but including more samples provides no further advantage once a sufficient number of samples are included.

**Table 1:** Median posterior values and 95% credible intervals for the parameters and past disease dynamics inferred from genealogies with different numbers of samples.

Sample Size	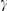	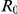	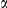	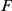	*Prevalence RMSD* *
40	2.36 [1.06, 4.18]	3.66 [1.21, 10.12]	0.25 [0.09, 0.51]	0.56 [0.03, 1.78]	244.77
100	2.94 [2.28, 3.95]	8.74 [3.97, 10.71]	0.19 [0.10, 0.32]	0.14 [0.02, 0.51]	93.23
200	3.05 [2.68, 3.39]	10.02 [9.37, 10.41]	0.15 [0.11, 0.21]	0.016 [0.001, 0.092]	39.55
400	3.00 [2.71, 3.29]	10.03 [8.42, 10.43]	0.16 [0.12, 0.20]	0.026 [0.001, 0.141]	73.89
**True Value**	3.00	10.00	0.16	0.012	

*Root Mean Squared Deviation (RMSD) for the prevalence was calculated using the deviation of the median of the posterior density from the true value summed over all time points.

Finally, we combined the simulated time series and genealogy to illustrate the ability of particle MCMC to be used with both sources of data. In Figure 2K–P, we show the posterior densities of the parameters when inferred from both the time series and a genealogy. In Figure 3C, we show the series of posterior densities for the latent variable *I* over time inferred from both the time series data and the genealogy. As shown, including the genealogy along with the time series data considerably reduces the uncertainty in both the estimates of the process model parameters and the past prevalence of the disease.

## Discussion

The framework we have developed extends phylodynamic inference in two major ways. First, stochastic state-space models that consider the biological processes driving population dynamics can be used instead of simple parametric or nonparametric demographic models when inferring past population dynamics. This also allows for key epidemiological parameters to be estimated directly from genealogies. Second, our approach allows for other sources of data such as time series to be considered along with a genealogy when inferring parameters and past population dynamics. Using a particle MCMC algorithm to fit a stochastic SIR model to simulated genealogies and time series data, we found that key epidemiological parameters as well as the past prevalence of the disease could be accurately estimated from genealogies with or without accompanying time series data.

While particle MCMC is computationally expensive because of the need to simulate particle trajectories each MCMC step, we believe it represents a good choice for the purposes of phylodynamic inference. First, particle MCMC allows for efficient MCMC sampling of model parameters and latent variables from their posterior densities even with high-dimensional, nonlinear SSMs. Secondly, particle MCMC is flexible in terms of the form of the SSMs that can be used. Because the particle filtering algorithm used in particle MCMC can be used to approximate the likelihood of the model through simulations, there is no need for an analytical likelihood function. Taken together, this allows for almost any infectious disease model to be used as long as particle trajectories can be simulated from the process model and an observation or coalescent model can be specified [23], [31], [32]. For example, several researchers including us here have used particle filtering methods to fit stochastic, continuous-time dynamic models to time series, even though observations occur only at discrete time points [25], [37], [38]. Finally, particle MCMC allows for flexibility in terms of the types and structure of the data. As we have shown, fitting dynamic models to different sources of data is straightforward since only the particle weighting scheme needs to be modified. We therefore believe that the computational cost of particle MCMC is outweighed by its flexibility and ease of implementation for most practical purposes in phylodynamics. Still, the efficiency of other statistical methodologies such as approximate Bayesian computation (ABC, see [39]) should be compared against particle MCMC in the future to see if the computational overhead of conducting phylodyamic inference with complex models can be reduced.

The particle MCMC approach described here is also able to incorporate different forms of stochasticity, which is essential for fitting the variation, or over-dispersion, present in real disease data. For simplicity, we only included environmental noise in the transmission process – random variation in the rate at which transmission events occur due to external factors like climatic fluctuations. However, other forms of stochasticity could also be included such as demographic stochasticity – random variation in the timing of demographic events such as the birth and death of individuals. We did not consider demographic stochasticity because it involves event-driven simulation approaches that are much more computationally expensive than the Euler-Maruyama algorithm we used. However, for small populations where demographic stochasticity can play an important dynamical role, other simulation methods could be employed within the particle filtering algorithm. For example, Breto *et al.* [23] recently introduced a simulation method that can include both environmental and demographic stochasticity. While what form of stochasticity is appropriate will be system-dependent, the need for statistical methods that include stochasticity when fitting models to disease data has been demonstrated repeatedly [22], [23], [37]. The particle MCMC approach therefore offers an advantage over other methods for phylodynamic inference that can only be used to fit deterministic models.

The ability to accurately infer past population dynamics or model parameters from genealogies ultimately depends on how sequences are sampled. Since we were primarily concerned with statistical methodology, we did not extensively explore different sampling strategies and simply considered the case where sequences are sampled randomly over time. However, we did find that only a moderate number of sequences are necessary to obtain reliable parameter estimates. Even when the sampling rate was as low as 10 samples per year, reliable estimates were obtained. Likewise, extremely large sample sizes did not significantly improve estimates, suggesting phylodynamic inference can be conducted without extensive sampling over time. Furthermore, even fewer samples may be necessary if sequences are sampled strategically. For example, in a simulation study, Stack *et al.* [40] found that accurate estimates of past population dynamics could be obtained using a variety of sampling protocols and that especially reliable estimates could be obtained if sequences are sampled towards the end of an epidemic rather than at the beginning of an epidemic. Our phylodynamic inference framework should therefore be able to give reliable estimates even if the sampling effort is not uniformly high over time.

We were also interested in when including the information contained within a genealogy alongside of time series date could improve estimation. At the most basic level, considering a genealogy where the coalescence times are known without error provides additional information in that the timing of coalescence events provides information about when transmission events occurred that is not present in temporally aggregated case report data. One could even imagine that knowing the complete genealogy of infections in the population would be preferable to having perfect case report data, since the exact times of infection will still not be known. In practice, we found that considering the genealogy alongside of time series data only significantly improved our estimates if there was observation error in the time series data. For example, the parameters estimated from the time series data with and without the genealogy in Figure 2 were done with a moderate level of observation error in the mock time series data. However, from our own experience, including the genealogy when there were low levels of observation error in the mock time series data did not significantly improve our estimates (results not shown). We therefore suspect that it will be helpful to include genealogical data only when the observed time series data are relatively uninformative about the true disease dynamics, such as when there is large degree of error in the case report data or when case report data are missing. Genealogies may also aid inference if aspects of the population dynamics such as periodicity or other long-term trends in disease dynamics are obscured by changes in reporting practices.

While we have shown that it is possible to fit complex population dynamic models to simulated genealogies, several challenges remain before this approach can be routinely applied to real data sets. First, while we conditioned our inference on knowing the true genealogy without error, the genealogy will have to be inferred from sequence data in any application of our method. Our uncertainty as to the true topology of the genealogy and the inferred coalescence times will then have to be considered. Fortunately, existing phylogenetic software packages like BEAST allow us to sample from the posterior distribution of trees so as to effectively integrate out phylogenetic uncertainty [41]. Furthermore, programs like BEAST also use an MCMC framework making it possible to estimate population dynamic parameters, the genealogy and the associated molecular evolutionary parameters together in a single MCMC framework by alternately sampling from the appropriate posterior densities.

Another challenge lies in formulating appropriate models for relating population dynamics to the reconstructed genealogy. The coalescent model we used may not be appropriate for all infectious diseases, just as the simple SIR model we used will not be adequate to describe the population dynamics of all diseases. For one, our coalescent model assumes neutrality with no phenotypic variation in the pathogen population, but real populations will be structured into multiple competing strains with varying antigenicity, pathogenicity and replication rates. Beyond selection, the natural history of a disease and heterogeneities due to population subdivision or contact structure can also have profound effects on genealogies [42], [43]. Likewise, sequence samples will often not be sampled randomly as assumed under standard coalescent models, leading to potential ascertainment biases if nonrandom sampling is not incorporated into coalescent models. However, the framework for phylodynamic inference presented here is extremely flexible and can be modified to accommodate more realistic population dynamic and coalescent models to account for these complications. For example, it should be possible to derive coalescent expressions for models with individual heterogeneity in infectivity and for SEIR models where infected individuals enter an exposed class before becoming infectious. Finally, when there are discrepancies between the disease dynamics inferred from genealogies and those observed in case report data, the ability to test different population dynamic and coalescent models in a coherent statistical framework will allow us to consider alternative hypotheses for what caused these discrepancies. This in turn should help improve our understanding of the complex ecological and evolutionary processes driving population dynamics — the central goal of phylodynamics [44], [45].

## Supporting Information

Text S1 Pseudo-code for particle MCMC.
(DOC)

Text S2 A description of the specific particle MCMC implementation used for the SIR epidemiological model.
(DOC)
