# Supplementary material for: Inference for Nonlinear Epidemiological Models Using Genealogies and Time Series
Source: PLoS Comput Biol. 2011 Aug 25;7(8):e1002136. doi: 10.1371/journal.pcbi.1002136 (PMC3161897; doi:10.1371/journal.pcbi.1002136)
Supplement: Text S1 — Pseudo-code for particle MCMC. (DOC) [file pcbi.1002136.s001.doc]

**Text S1:** Pseudo-code for particle MCMC

We divide the pseudo-code below into two parts: algorithm 1 implements the MCMC component of the particle MCMC method, calling on algorithm 2, which implements the particle filter component of the particle MCMC method.

**Algorithm 1:** the MCMC component of particle MCMC

In the following algorithm, $q$ is the vector containing all model parameters, $x_{1:T}$ are the latent variables and $z_{1:T}$ are the observed data. *m* indexes the MCMC iterations from 1 to *M*.

**Step 1:** Initialize MCMC

1. Set m = 0.
2. Set $q(0)$ arbitrarily
3. Run particle filter (Algorithm 2) to sample $x_{1:T}(0)$ from and obtain the marginal likelihood estimate $\hat{p}(z_{1:T}|q(0))$.

**Step 2:** Run MCMC

**For** *m = 1 to M*

1. Sample $q*$ from a proposal density $q(q*|q(m-1))$.
2. Run Algorithm 2 to sample$x_{1:T}*$ from and obtain the marginal likelihood estimate $\hat{p}(z_{1:T}|q*)$.
3. With probability

,

set $q(m)=q*$, $x_{1:T}(m)=x_{1:T}*$ and ;

**else** set $q(m)=q(m-1)$, $x_{1:T}(m)=x_{1:T}(m-1)$ and .

**End for**

**Algorithm 2:** the particle filter component of particle MCMC

In the following algorithm, $q$ is the vector containing all model parameters, $x_{1:T}$ are the latent variables and $z_{1:T}$ are the observed data. *t = 1 to T* are the observation times.$j$ = *1* to *N* are the particle indices. For example, $x_{t}^{j}$ represents the state of particle *j* at time *t*. The notation $A_{t}^{j}$ is used to track the ancestry of particles backward in time, such that $A_{t-1}^{j}$ represents the parent index of particle *j* at time *t*.

**Step 1:** Initialize particle filter at time *t = 1*

1. Set $x_{1}^{j}$ to initial values for all particles.
2. Assign unnormalized particle weights

**.**

1. Normalize the particle weights

.

**Step 2:** Run particle filter

**For** *t =2 to T*

1. Resample particles by sampling parent particle indices *k* according to their weights, such that

.

(b) Set for all particles.

(c) Propagate particles by simulating from the process model

to the next observation time t.

(d) Set for all particles.

(e) Assign incremental, unnormalized particle weights

**.**

(f) Normalize particle weights

.

**End for**

**Step 3:** Estimate marginal likelihood

**,**

where

.

**Step 4:** Sample $x_{1:T}*$ from by tracing the lineage of one particle trajectory backwards through time

1. Sample a particle index *l_T_* such that

.

**For** *t = T* back to *2*

(b) set $l_{t-1}$ equal to its parent index $A_{t-1}^{l_{t}}$.

**End for**

(c) Set .
