# Supplementary material for: Inference for Nonlinear Epidemiological Models Using Genealogies and Time Series
Source: PLoS Comput Biol. 2011 Aug 25;7(8):e1002136. doi: 10.1371/journal.pcbi.1002136 (PMC3161897; doi:10.1371/journal.pcbi.1002136)
Supplement: Text S2 — A description of the specific particle MCMC implementation used for the SIR epidemiological model. (DOC) [file pcbi.1002136.s002.doc]

**Text S2:** A description of the specific particle MCMC implementation used for the SIR epidemiological model

For all particle MCMC runs whose results are shown in the main text, the Markov chain was run until convergence was reached for all parameters and latent variables. Convergence was checked by visually inspecting trace plots of parameter values over the MCMC steps. For the results shown in the main text, the particle MCMC was run for at least 20,000 MCMC steps. Initial values for the parameters in $q$ were arbitrarily assigned. Uniform priors were placed on all parameters. A multivariate normal proposal density was used to propose new values for the parameters in $q$,

,

where $S$ is the covariance matrix. A truncated multivariate normal density was used where parameters needed to be constrained to only take on values in a given range. To facilitate mixing, a covariance matrix $S$ for the proposal density was chosen after a preliminary MCMC run so that correlations between the accepted parameter values could be considered.

For the particle filter, the number of particles *J* was chosen based on our experience with different particle population sizes. While particle MCMC can provide an exact approximation to the posterior densities of the parameters and latent variables using any number of particles, in practice we found that the choice of *J* has drastic effects on the efficiency of the MCMC. Specifically, a small number of particles will lead to a large variance in the Monte Carlo estimates of the marginal likelihood provided by the particle filter, which can lead to poor mixing in the MCMC. On the other hand, simulation time increases with the number of particles used. We therefore chose to use an intermediate number of particles, with *J* = 200 for all results shown in the main text.

We also modify the general particle filtering algorithm (Algorithm 2) presented in Text S1 to accommodate the structure of our time series data. Specifically, since we consider temporally aggregated incidence data, it is not possible to compute the likelihood of the observed data at *t=1* without tracking the cumulative number of new infections in the previous time interval. We therefore modify Step 1 in Algorithm 2 so that particles are initiated at *t = 0* instead of *t = 1*. At *t=0*, we set $x_{0}^{j}$ equal to the initial values for all particles, which can either be fixed or estimated as a component of $q$. All particles are then assigned equal unnormalized particle weights so that the normalized particle weights are $W_{0}^{j}=\frac{1}{N}$ for all *j*. In step 2, we then run the particle filter from *t =1* to *t =T*. Likewise in step 4 we move backward from *t = T* to *t = 1* to accommodate the additional time point at *t=0*,

All code for the particle MCMC algorithm and the code used to simulate mock data was written and run in Matlab version 7 and is available from our website: www.biology.duke.edu/koellelab. The particle MCMC code can also be run in Octave. We hope to have an R version of our particle MCMC code available soon.
